# Distinct Profiles of CD163-Positive Macrophages in Idiopathic Interstitial Pneumonias

**DOI:** 10.1155/2018/1436236

**Published:** 2018-02-04

**Authors:** Masahiro Yamashita, Ryoko Saito, Shinji Yasuhira, Yuh Fukuda, Hironobu Sasamo, Tamotsu Sugai, Kohei Yamauchi, Makoto Maemondo

**Affiliations:** ^1^Department of Pulmonary Medicine, Allergy and Rheumatology, Iwate Medical University School of Medicine, Morioka, Japan; ^2^Department of Pathology, Tohoku University Graduate School of Medicine, Sendai, Japan; ^3^Department of Cancer Biology, Iwate Medical University, Shiwa, Japan; ^4^Division of Diagnostic Pathology, Itabashi Chuo Medical Center, Tokyo, Japan; ^5^Department of Analytic Human Pathology, Nippon Medical School, Tokyo, Japan; ^6^Department of Pathology, Iwate Medical University School of Medicine, Morioka, Japan

## Abstract

**Background:**

The types of cells most significantly linked to individual subtypes of idiopathic interstitial pneumonias (IIPs) remain unclear. Few studies have examined CD163^+^ macrophages in IIPs.

**Objective:**

We retrospectively aimed to immunohistochemically characterize the CD163^+^ macrophages in IIPs.

**Methods:**

Paraffin-embedded lung tissue samples were obtained from 47 patients with IIPs, including idiopathic pulmonary fibrosis (IPF), idiopathic nonspecific interstitial pneumonia (NSIP), and cryptogenic organizing pneumonia (COP), and 12 normal controls were immunohistochemically analyzed, using primary antibodies against CD68 and CD163 as indicators of pan and M2 macrophages, respectively.

**Results:**

CD68^+^ macrophage density was significantly increased in the 3 subtypes of IIPs relative to that in the control group, although no difference was detected within the different IIPs. CD163^+^ macrophage density was significantly increased in NSIP and COP samples relative to that in IPF samples. The density ratio of CD163^+^ macrophages to CD68^+^ macrophages was significantly decreased in IPF/UIP samples relative to that in the others, while the densities in NSIP and COP were significantly higher than those in control cases.

**Conclusion:**

CD163^+^ macrophages show distinct profiles among IIPs, and the standardized numerical density is decreased in IPF cases that have poor prognoses.

## 1. Introduction

Idiopathic interstitial pneumonias (IIPs) are a heterogeneous group of acute and chronic disorders with varying degrees of inflammation and fibrosis; the etiologies of which are unknown [1]. Idiopathic pulmonary fibrosis (IPF)/usual interstitial pneumonia (UIP), nonspecific interstitial pneumonia (NSIP), and cryptogenic organizing pneumonia (COP) have gained attention because of their relatively high incidences. The prognoses of IPF/UIP are poor relative to those of the latter two types, and different pathological features enable discrimination of these conditions [1]. IPF/UIP is histopathologically characterized by temporal heterogeneity in the degree of interstitial fibrosis in the alveolar septa, including in normal regions and severe fibrotic areas [2, 3]. The presence of intraluminal fibrotic lesions, known as fibroblastic foci, is associated with the prognosis of IPF/UIP [3]. NSIP is mainly characterized by a dense or loose interstitial fibrosis with a uniform appearance. COP primarily shows intraluminal fibrotic involvement with a patchy distribution [4, 5]. The background lung architecture is well preserved in COP and NSIP, while a honeycomb lung represents a terminal status of tissue remodeling in IPF/UIP [2–5]. Importantly, the types of cells most significantly linked to differences in the pathogenesis and prognoses among the subtypes of IIPs remain unclear.

Macrophages constitute a heterogeneous population of cells of the innate immune system and display a variety of functions [6]. This functional diversity of macrophages develops from the response to local microenvironmental signals that allow them to adapt to this local environment, with the cells typically represented as M1 and M2 types of populations [7]. M1 macrophages are characterized by the ability to produce proinflammatory mediators, which is associated with phagocytosis, killing of microorganisms, and tissue injury [8–10]. In contrast, M2 macrophages have an anti-inflammatory effect, which is linked to the phagocytosis of apoptotic cells, tissue repair, and fibrosis [11–15]. Particularly, an increasing number of studies have revealed a significant association between fibrotic diseases and macrophages with positivity for CD163, an endocytic receptor for heme and ferroportin and M2 marker [16–20].

We hypothesized that subpopulations of macrophages are represented differently among the subtypes of IIPs, as macrophages exhibit plastic responses to different microenvironments. Different types of subpopulations indeed participate in individual processes corresponding to the development and repair of fibrosis models [21, 22]. However, limited information is available regarding the subpopulations of macrophages in IIPs. In the present study, we immunohistochemically characterized CD68^+^ and CD163^+^ macrophages in the three subtypes of IIPs: IPF/UIP, NSIP, and COP. CD68 is a single-chain glycoprotein of 110 kD expressed predominantly on the lysosomal membrane of myeloid cells and is thought to be a pan-macrophage marker [23]. Our findings revealed interesting characteristics for the counting of CD163^+^ macrophages among IIPs.

## 2. Materials and Methods

### 2.1. Materials

A total of 87 patients who underwent video-assisted thoracoscopic surgery biopsy for the diagnosis of interstitial pneumonia at Iwate Medical University Hospital (Morioka, Japan) and Tohoku University Hospital (Sendai, Japan) from 2000 to 2010 were selected for the study. Of these, 40 patients were excluded from the study because of diagnoses of interstitial pneumonias other than IIPs, such as collagen vascular diseases or hypersensitivity pneumonitis. The remaining 47 patients were retrospectively diagnosed with idiopathic interstitial pneumonias based on multiple disciplinary discussions, as per international consensus criteria [24], and consisted of 23 with IPF/UIP, 17 with NSIP, and 7 with COP. No patient received treatment before surgical biopsy. After diagnosis, treatment was administered according to the guidelines in effect at the time of the diagnosis [24]. The mean follow-up of patients alive at the endpoint of analysis was 56.8 months. For the control lungs, normal regions distant from the cancer lesions in lung specimens obtained from 12 patients with preinvasive lung adenocarcinoma were used. These specimens were obtained from the archives of the Departments of Pathology at Iwate Medical University and Tohoku University Hospital, the Ethical Committees which approved the use of all samples in this study (IRB, H24-170, and 2014-1-446). The requirement for informed consent was waived because of the retrospective nature of this study.

### 2.2. Immunohistochemistry

As primary antibodies, mouse anti-human CD68 (clone PGM-1, DAKO, Glostrup, Denmark, dilution 1 : 50), mouse anti-human CD163 (clone 5C6, BMA Biomedicals, Augst, Switzerland, dilution 1 : 200), and anti-human CD163 (clone EDhu1, Serotec, Cambridge, UK, dilution 1 : 200) antibodies were used. As negative controls for each antibody, normal mouse IgG1 (Dako) and normal rabbit serum (Vector Labs, Burlingame, CA, USA) were used. The detailed protocol used for immunohistochemistry analysis has been previously described [25].

### 2.3. Morphometric Analysis

The numerical density of CD68^+^ [*N*_A_(CD68)] and CD163^+^ [*N*_A_(CD163)] mononuclear cells was counted at 200-fold magnification in 20 randomly sampled fields per slide [25]. Alveolar and interstitial macrophages were separately counted. Areas corresponding to 1–3 degrees based on Ashcroft's fibrotic score were estimated to compare the 3 conditions, as IPF samples exhibit temporarily heterogeneous lesions [26]. The numerical density of macrophages was standardized according to the interstitial number densities [*N*_A_(int)], which were measured by point counting methods using a grid [25, 27]. In IPF samples, the alveolar and interstitial numerical density of CD68^+^ [*N*_A_(CD68)] and CD163^+^ [*N*_A_(CD163)] macrophages was estimated, which were divided into 2 fibrotic grades including mild and severe lesions. Densities were standardized by air space and interstitial area density as [*A*_A_(Air)] and [*A*_A_(int)], respectively [28]. Lung specimens were obtained from multiple lobes as far apart as possible. Morphometric analyses were performed in individual lobes, and average values were used as representative data of each patient. Morphometric examinations were performed independently by two pathologists (R.S., T.S.).

### 2.4. Pulmonary Function Tests

The forced vital capacity, forced expiratory volume in 1 s, and diffuse capacity of the lung for carbon monoxide were measured according to American Thoracic Society guidelines [29]. These values were also expressed as percentages of the predicted normal values calculated according to sex, weight, and age [30].

### 2.5. Statistical Analysis

Statistical significance was evaluated by one-way analysis of variance followed by Dunnett test or Fisher's exact test. Receiver operating characteristic (ROC) curves were plotted for standardized numerical density of CD163^+^ macrophages and differential diagnosis between IPF and NSIP. A diagnostic test with an area under the curve (AUC) above 0.75 was regarded as contributive [31]. A *p* value less than 0.05 was considered to indicate statistical significance. Statistical analyses were performed using SPSS Statistics software (SPSS Inc., Chicago, IL, USA).

## 3. Results

### 3.1. Patient Characteristics

Patient characteristics are shown in Table 1. Patients with NSIP were younger than those with IPF/UIP (*p* < 0.05).

### 3.2. Morphological and Morphometric Analyses of CD68 Macrophages in IIPs

**Figure 1:**
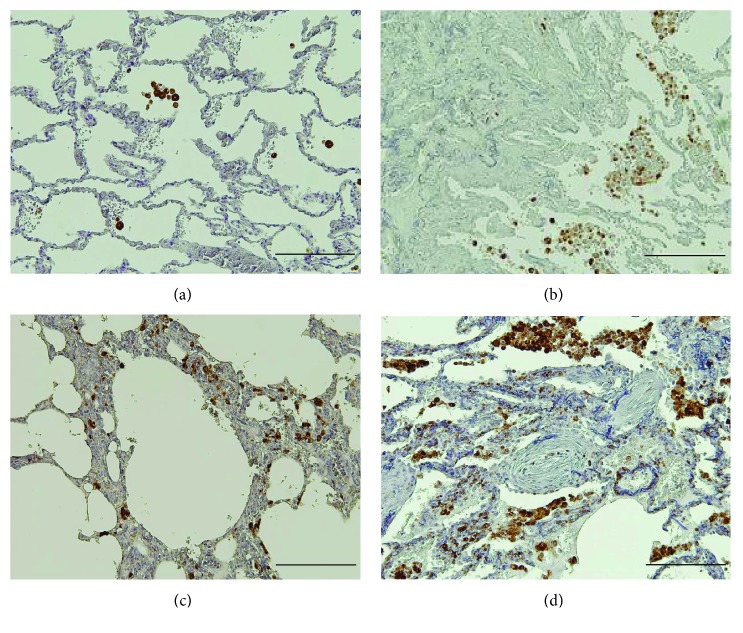
Immunohistochemical analysis of CD68 expression in IIPs. (a) CD68^+^ macrophages (*Brown*) were scattered in the alveolar space of normal control lungs. Numerous CD68^+^ macrophages were observed in the alveolar space in IIPs, including IFF/UIP (b), NSIP (c), and COP (d). In the interstitium, CD68^+^ macrophages were observed in NSIP and COP, but barely detectable within the intraluminal fibrosis in COP. Resorcin-fuchsin and hematoxylin were used as counterstains. Scale bar, 200 *μ*m.

**Figure 2 fig2:**
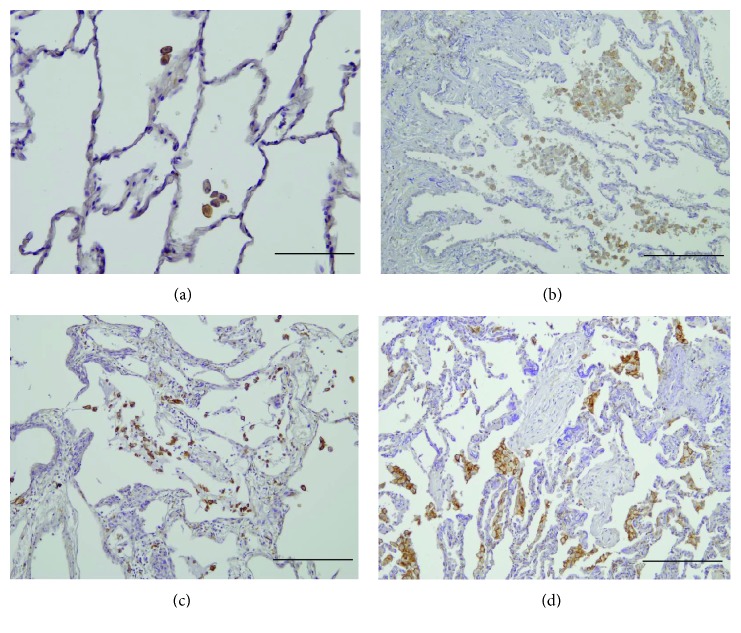
Immunohistochemical analysis of the expression of CD163 in IIPs. CD163^+^ macrophages (*Brown*) are observed to be scattered in normal control lungs (a). In IPF/UIP, numerous alveolar macrophages show weak or no expression of CD163 (b). Numerous CD163^+^ macrophages are observed predominantly in alveolar space of NSIP (c) and COP (d) and in interstitium too. CD163^+^ macrophages were rarely detected within fibroblastic foci in IPF/UIP and the intraluminal fibrosis in COP. Resorcin-fuchsin and hematoxylin were used as counterstains. Scale bar: (a) 100 *μ*m and (b–d) 200 *μ*m.

**Figure 3:**
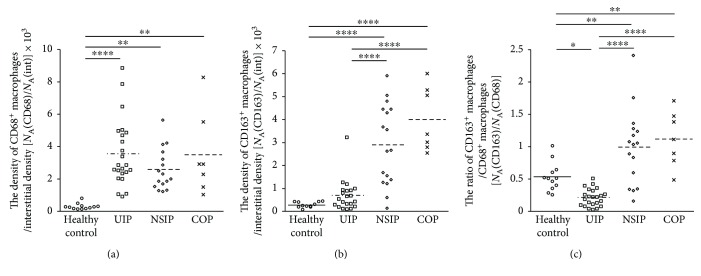
Comparison of CD68^+^ and CD163^+^ macrophage densities among the four groups. The numerical densities of CD68^+^ macrophages standardized by interstitial density [*N*_A_(CD68)/*N*_A_(int)] were significantly increased in IPF/UIP, NSIP, and COP relative to those in normal control lungs (a). The numerical densities of CD163 macrophages [*N*_A_(CD163)/*N*_A_(int)] were significantly increased in NSIP and COP relative to those in normal control lungs and IPF/UIP (b). The ratio of CD163^+^ macrophages to CD68^+^ macrophages was significantly increased in IPF/UIP and normal control lungs relative to those in the other 2 groups (c) [*N*_A_(CD163)/*N*_A_(CD68)]. The values of the numerical densities described in the figures represent actual values multiplied by 10^3^. ^∗^ *p* < 0.05, ^∗∗^ *p* < 0.01, and ^∗∗∗∗^ *p* < 0.0001.

We immunohistochemically characterized CD68^+^ and CD163^+^ macrophages in the 4 groups, including normal control lungs, IPF/UIP, NSIP, and COP (Figures 1 [Fig fig2]–3). CD68^+^ macrophages were scattered in the control lungs (Figure 1(a)), while high numbers were observed in every type of IIP (Figures 1(b)–1(d)). Numerous CD68^+^ macrophages were observed within airspace neighboring mild and severe fibrotic lesions (Supplementary Figure E1A and B), but were undetectable within fibroblastic foci of IPF/UIP (Supplementary Figure E1C). The numerical density of CD68^+^ macrophages was significantly increased in the 3 types of IIPs relative to that in the control, although no difference was observed among the 3 disease groups (Figure 3(a)). While categorizing fibrotic lesions in IPF/UIP into 2 severity grades, CD68^+^ macrophages were detected in both lesions (Supplementary Figure E1C and D). However, the standardized density of CD68^+^ alveolar macrophages [*N*_A_(CD68)/*A*_A_(air)] showed significantly higher levels in severe lesions relative to those in mild lesions, although no difference was detected in interstitial density [*N*_A_(CD68)/*A*_A_(int)] (*p* < 0.0001) (Supplementary Figure E2A and B).

### 3.3. Morphological and Morphometric Analyses of CD163^+^ Macrophages in IIPs

CD163^+^ macrophages showed a scattered distribution in normal control samples (Figure 2(a)). In the airspace neighboring the mild lesions of IPF/UIP, numerous macrophages showed weak or no expression of CD163, although a few CD163^+^ macrophages were observed (Figure 2(b), Supplementary Figure E1E). In interstitial lesions of IPF/UIP, very few CD163^+^ macrophages were detected (Supplementary Figure E1G and H). In contrast, these cells were abundant in NSIP and COP (Figures 2(c) and 2(d)). The numerical density of CD163^+^ macrophages was significantly increased in NSIP and COP relative to those in the control group and IPF/UIP (Figure 3(b)). Although CD163^+^ mononuclear cells locally formed cluster aggregation in the airspace neighboring severe fibrotic lesions, there was no difference in the standardized numerical density of CD163^+^ alveolar macrophages between the airspaces adjacent to mild and severe fibrotic lesions (Supplementary Figure E1E and F and Figure E2C). There was no difference in the numerical densities of CD163^+^ interstitial macrophages between the two severity grades of lesions of IPF/UIP (Supplementary Figure E2D). In the present study, although the data are represented as the results obtained with anti-CD163 antibody (clone EDhu1), both antibodies of clone 5C6 and EDhu1 showed similar results.

### 3.4. Density Ratio of CD163^+^ Macrophages to CD68^+^ Macrophages

The density ratio of CD163^+^ macrophages to CD68^+^ macrophages was significantly decreased in mild lesions of IPF/UIP relative to that in the others, although the densities in NSIP and COP were significantly higher than those in control cases (Figure 3(c)). The significant difference in the ratio was also observed in alveolar and interstitial macrophages (Supplementary Figure E3).

### 3.5. Differences between Nonsmokers and Smokers

We also explored the influence of smoking on CD68^+^ and CD163^+^ macrophage densities in normal control, IPF/UIP, and NSIP cases. We did not determine the effects of smoking in patients with COP because the number of patients was too less. There was no difference in CD68^+^ macrophage densities between nonsmokers and smokers in every condition (Supplementary Figure E4). However, CD163^+^ macrophage density was significantly lower in smokers with NSIP, and the ratio of CD163^+^ macrophages to CD68^+^ macrophages showed a decreasing trend in smokers with IPF/UIP (Supplementary Figure E4F and H).

### 3.6. Diagnostic Value of CD163^+^ Macrophage Densities in Differentiation between IPF/UIP and NSIP

**Figure 4:**
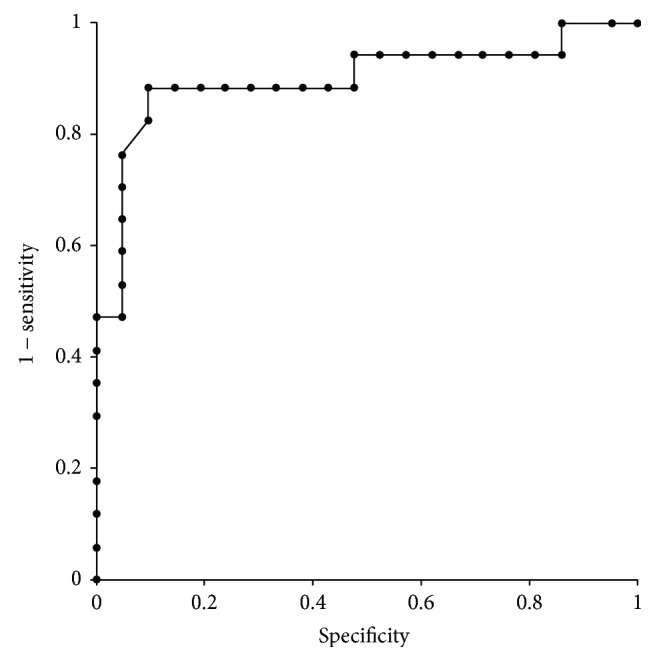
The results of receiver operating characteristic (ROC) curve analysis. The value of ROC-area under the curve shows 0.898 (95% confidence interval, CI, 0.783–1.000) for the diagnostic differentiation between IPF/UIP and NSIP. A cut-off level below 12.0 in total numerical density of CD163^+^ macrophages yielded a sensitivity of 90.5% (95% CI = 78.2–96.2%) and specificity of 88.2% (95% CI = 73.1–95.3%) for the diagnosis of IPF/UIP.

We explored the diagnostic value of the numerical density of CD163^+^ macrophages in the differentiation between IPF/UIP and NSIP, using ROC analysis. The total numerical density of CD163^+^ macrophages showed an ROC-AUC value of 0.898 (95% confidence interval, CI, 0.783–1.000) for the differentiation (Figure 4). A cut-off level of 12.0 in total numerical density of CD163^+^ macrophages yielded a sensitivity of 90.5% (95% CI = 78.2–96.2%) and specificity of 88.2% (95% CI = 73.1–95.3%). Moreover, we evaluated the relation between the response to the treatment and numerical density of CD163^+^ macrophages. No statistically significant relation was detected in any group; the coefficient of correlation was determined to be 0.49 in patients with NSIP and COP who received corticosteroids for treatment.

## 4. Discussion

In the present study, we found that the numerical density of CD68^+^ macrophages was higher in the 3 types of IIPs relative to that in the normal control lungs, while CD163^+^ macrophages density was higher in NSIP and COP than in IPF/UIP. The density ratio of CD163^+^ macrophages to CD68^+^ macrophages was significantly lower in IPF/UIP relative to those in the other 3 groups, while the ratios in COP and NSIP were significantly higher relative to that in the normal control lungs.

Very limited information is available regarding the characterization of CD163^+^ macrophages in IIPs. Wojtan et al. estimated the proportion of CD163^+^ macrophages in bronchoalveolar lavage fluids by immunocytochemistry [32]. The proportion of CD163^+^ macrophages did not differ between IPF/UIP and NSIP, which is inconsistent with our results. However, as they did not use pan-macrophage markers, the proportions represented in their study are unclear. In addition, it is difficult to draw conclusions regarding the association between IIPs and CD163^+^ macrophages in their study, as they used a very small sample size of 6 patients with IPF/UIP and 8 with NSIP.

There are two mechanistic possibilities explaining how the higher ratio of CD163^+^ macrophages to CD68^+^ macrophages is related to interstitial pneumonia, although we could not determine the pathogenic roles of the macrophages in IIPs in the present study. The first possibility is that CD163^+^ macrophages have a protective role against tissue injury associated with IIPs. Ye et al. reported decreased expression of heme oxygenase-1 in alveolar macrophages in idiopathic pulmonary fibrosis patients [33]. CD163 is a scavenger receptor for the heme-haptoglobin complex, which reduces the toxicity of heme-oxygenase. Our findings and those of previous studies suggest a protective role of CD163^+^ macrophages against IIPs. In contrast, the second possibility is that CD163 macrophages accelerate fibrosis in non-IPF/UIP, which has a relatively better prognosis, such as NSIP and COP. Christmann et al. reported that mRNA expression of CD163 was upregulated in the lung specimens obtained from patients with systemic sclerosis-associated interstitial lung diseases (SSc-ILD), which mainly consisted of NSIP, and that CD163 gene expression levels were correlated with the progression of fibrosis based on HRCT [34]. Mathai et al. reported that mRNA expression of CD163 was upregulated in monocytes in the peripheral blood of patients with SSc-ILD relative to that in healthy controls [35].

Interestingly, our results showed that CD163^+^ macrophages were associated with IPF/UIP to a lesser extent, although increasing evidence suggests a positive association between CD163^+^ macrophages and fibrogenic conditions. It is very important to consider the possibility that the development of fibrotic lesions in IPF/UIP does not depend on acceleration by CD163^+^ macrophages, and it remains to be elucidated which types of cells most significantly regulate the prolonged activity of myofibroblasts in IPF/UIP.

It has been reported that smoking influences macrophage polarization [36]. In the present study, we examined the influence of smoking on CD68^+^ and CD163^+^ macrophage densities in normal control lungs, IPF/UIP, and NSIP. CD163^+^ macrophage density was decreased in NSIP patients who smoked, and the ratio of CD163^+^ macrophages to CD68^+^ macrophages showed a decreasing trend in IPF/UIP patients who smoked. The comparative data of CD163 macrophage density among the 3 groups were unlikely to be biased by smoking because the ratios of patients with smoking were equivalent in the 3 conditions.

In the clinical setting, NSIP is diagnostically differentiated from IPF/UIP, and histopathologic analysis is routinely required for diagnosis. However, it is not always easy to differentiate between the two conditions, and multiple disciplinary discussions are often required to determine the diagnosis [37, 38]. No information is available regarding a diagnostic marker for the differentiation of IIP subtypes. In the present study, the high value of the ROC-AUC suggests the potential of CD163^+^ macrophage density as a useful differential marker.

The present study has some limitations. The first limitation is its retrospective nature. It is unlikely that there was selection bias in patients with IIPs because we consecutively enrolled patients at both institutes. Second, we could not estimate the numerical densities of CD68^+^ and CD163^+^ macrophages in multiple lobes in all patients with IIPs. However, no difference in the numerical densities of CD68^+^ and CD163^+^ macrophages was observed between the upper and lower lobes in each group (data not shown). Third, the study population was relatively small. Further studies on a larger cohort of patients are needed to validate the diagnostic value of CD163^+^ macrophage density in IIPs.

We clearly demonstrated the distinct profiles of CD163^+^ macrophage counts among the subtypes of IIPs. The lower ratio of CD163^+^/CD68^+^ macrophages was related to IPF/UIP, and CD163^+^ macrophages may be diagnostically useful markers for differentiating IIPs. Our results provide insight into the pathogenic and clinical perspectives of IIPs and may facilitate further investigations of the heterogeneity of macrophages in IIPs.

## Figures and Tables

**Table 1:** Characteristics of healthy volunteers and patients with IIPs.

	Healthy control	UIP	NSIP	COP	
*n*	12	23	17	7	*p* value
Age	64.3 ± 3.1	65.3 ± 1.6	54.3 ± 2.7	64.0 ± 3.9	0.015
Female (%)	6 (50.0)	10 (43.5)	8 (46.6)	2 (28.6)	n.s
Smoke (%)	6 (50.0)	12 (52.2)	5 (29.4)	4 (57.2)	n.s
Pulmonary function tests					
FVC (L)	3.03 ± 0.24	2.48 ± 0.19	2.55 ± 0.30	2.19 ± 0.23	n.s
FVC (%)	112.7 ± 3.0	83.3 ± 4.2	78.4 ± 7.2	68.3 ± 5.9	n.s
FEV/FVC (%)	77.8 ± 2.1	87.6 ± 2.0	85.0 ± 1.41	81.4 ± 1.9	n.s
DLco (%)	114.2 ± 7.6	73.7 ± 5.1	67.8 ± 6.9	63.4 ± 4.8	n.s
Lung specimens obtained					
Upper lobe (%)	6 (50.0)	5 (21.7)	1 (5.8)	1 (14.3)	n.s
Lower lobe (%)	6 (50.0)	7 (30.4)	3 (17.6)	3 (42.9)	n.s
Both lobes (%)	0 (0)	11 (47.8)	13 (76.5)	3 (42.9)	<0.001
Treatments					
Corticosteroid (%)	0 (0)	4 (17.4)	13 (74.5)	5 (71.4)	<0.001
Immunosuppresants (%)	0 (0)	2 (8.7)	5 (29.4)	0 (0)	n.s
Pirfenidone (%)	0 (0)	2 (8.7)	1 (5.9)	0 (0)	n.s
None (%)	12 (100.0)	16 (69.6)	3 (17.6)	2 (28.6)	<0.001

Data are shown as mean ± SD. Brackets represent percentage. IPF/UIP: idiopathic pulmonary fibrosis/usual interstitial pneumonia; NSIP: nonspecific interstitial pneumonia; COP: cryptogenic organizing pneumonia; FVC: forced vital capacity; FEV_1.0_: forced expiratory volume in 1 second; DLco: diffusing capacity of the lungs for carbon monoxide; n.s: no statistical significance.

## Supplementary Materials

Supplementary 1 Figure E1: immunohistochemical analyses of CD68 and CD163 expression in mild and severe fibrotic lesions of IPF/UIP. Many CD68^+^ macrophages with strong expression (brown) were observed in the airspaces neighboring mild (A) and severe (B) interstitial fibrotic lesions. CD68^+^ macrophages were scattered within mild (C) and severe (D) interstitial fibrotic lesions. CD68^+^ macrophages were not detected within fibroblastic foci (arrows in C). In contrast, numerous macrophages showed weak or no expression of CD163 in the alveolar space near mild fibrotic lesions, although few CD163^+^ macrophages were detected (E). In the alveolar space near severe fibrotic lesions of IPF/UIP, CD163^+^ macrophages showed cluster aggregation (F). CD163^+^ macrophages with weak expression (brown) were occasionally observed in mild (G) and severe (H) interstitial fibrotic lesions. Resorcin-fuchsin and hematoxylin were used as counterstains. Scale bar, 100 *μ*m.

Supplementary 2 Figure E2: comparison of CD68^+^ and CD163^+^ macrophage densities between mild and severe fibrotic grades of lesions in IPF/UIP. (A) A comparison of the numerical densities of alveolar CD68^+^ macrophages between airspaces near mild and severe fibrotic lesions in IPF/UIP. The numerical density of CD68^+^ alveolar macrophages standardized by airspace area density [*N*_A_(CD68)/*A*_A_(air)] in severe fibrotic lesions showed a significant increase relative to that in mild lesions. In contrast, no difference was detected in the numerical densities of CD68^+^ interstitial macrophages standardized by interstitial numerical density [*N*_A_(CD68)/*N*_A_(int)] (B). There was no difference in the numerical densities of CD163^+^ alveolar macrophages [*N*_A_(CD163)/*A*_A_(air)] and interstitial macrophages [*N*_A_(CD163)/*N*_A_(int)] between mild and severe lesions (C and D). The values of the numerical densities described in the figure represent actual values multiplied by 103. ^∗∗∗∗^ *p*<0.0001.

Supplementary 3 Figure E3: comparison of alveolar and interstitial densities of CD68^+^ and CD163^+^ macrophages among the 4 groups. The results of alveolar macrophages are similar to those of total macrophages in the case of CD68^+^ [*N*_A_(CD68)/*N*_A_(int)] (A), CD163^+^ macrophages [*N*_A_(CD163)/*N*_A_(int)] (B), and ratio of CD68^+^ macrophages to CD163^+^ macrophages [*N*_A_(CD68)/*N*_A_(CD163)] (C). The interstitial density of CD68^+^ macrophages [*N*_A_(CD68)/*N*_A_(int)] is similar to those of total macrophages, although CD68^+^ interstitial macrophages were not detected in 9 of 12 control cases (D). The results of CD163^+^ interstitial macrophages [*N*_A_(CD163)/*N*_A_(int)] are similar to those of alveolar and total macrophages (E). The interstitial ratio of CD68^+^ macrophages to CD163^+^ macrophages [*N*_A_(CD163)/*N*_A_(CD68)] showed a significant increase in IPF/UIP relative to that in the others (F). The values of numerical densities described in the figure represent actual values multiplied by 103. ^∗^ *p*<0.05, ^∗∗^ *p*<0.01, ^∗∗∗^ *p*<0.001, and ^∗∗∗∗^ *p*<0.0001.

Supplementary 4 Figure E4: comparison of numerical densities of CD68^+^ and CD163^+^ macrophages between nonsmokers and smokers. There was no difference in CD68^+^ and CD163^+^ macrophage densities and the ratio of CD163^+^ macrophages to CD68^+^ macrophages between nonsmokers and smokers in the normal control lungs (A–C). The ratio of CD163^+^ macrophages to CD68^+^ macrophages showed a decreasing trend in smoker patients with IPF/UIP relative to that in nonsmokers (F). A significant decrease was detected in smoker patients with NSIP relative to that in nonsmokers (H). The values of numerical densities described in the figure represent actual values multiplied by 103. ^∗^ *p*<0.05.
