# Supplementary figures and images for: Distinct Profiles of CD163-Positive Macrophages in Idiopathic Interstitial Pneumonias
Source: J Immunol Res. 2018 Feb 4;2018:1436236. doi: 10.1155/2018/1436236 (PMC5817286; doi:10.1155/2018/1436236)

## Slide 1
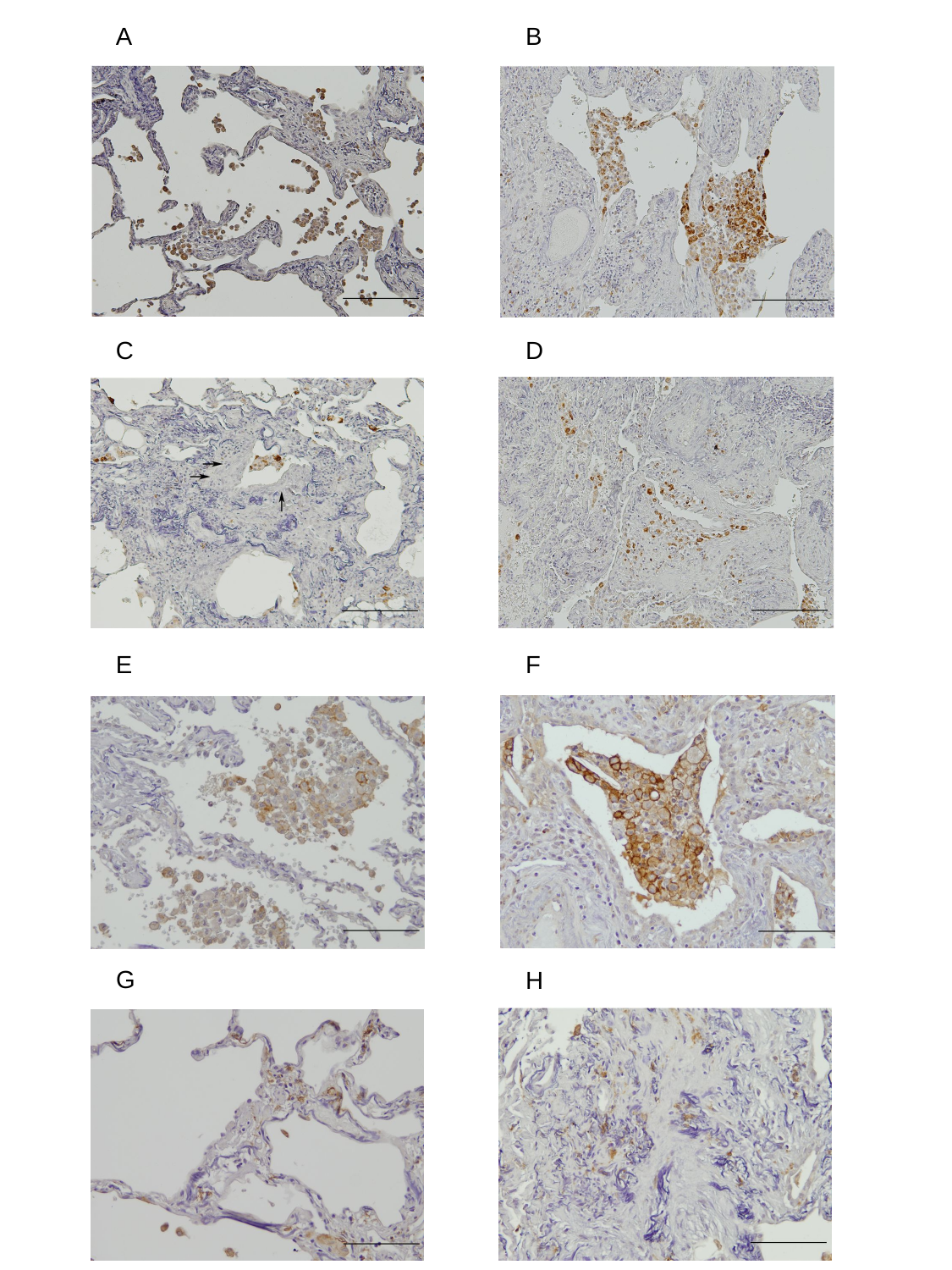

A
B
C
D
E
F
G
H

Supplement: Supplementary 1 — Figure E1: immunohistochemical analyses of CD68 and CD163 expression in mild and severe fibrotic lesions of IPF/UIP. Many CD68+ macrophages with strong expression (brown) were observed in the airspaces neighboring mild (A) and severe (B) interstitial fibrotic lesions. CD68+ macrophages were scattered within mild (C) and severe (D) interstitial fibrotic lesions. CD68+ macrophages were not detected within fibroblastic foci (arrows in C). In contrast, numerous macrophages showed weak or no expression of CD163 in the alveolar space near mild fibrotic lesions, although few CD163+ macrophages were detected (E). In the alveolar space near severe fibrotic lesions of IPF/UIP, CD163+ macrophages showed cluster aggregation (F). CD163+ macrophages with weak expression (brown) were occasionally observed in mild (G) and severe (H) interstitial fibrotic lesions. Resorcin-fuchsin and hematoxylin were used as counterstains. Scale bar, 100 μm. [file 1436236.f1.pptx]
