# Supplementary material for: Distinct Profiles of CD163-Positive Macrophages in Idiopathic Interstitial Pneumonias
Source: J Immunol Res. 2018 Feb 4;2018:1436236. doi: 10.1155/2018/1436236 (PMC5817286; doi:10.1155/2018/1436236)
Supplement: Supplementary 2 — Figure E2: comparison of CD68+ and CD163+ macrophage densities between mild and severe fibrotic grades of lesions in IPF/UIP. (A) A comparison of the numerical densities of alveolar CD68+ macrophages between airspaces near mild and severe fibrotic lesions in IPF/UIP. The numerical density of CD68+ alveolar macrophages standardized by airspace area density [N A(CD68)/A A(air)] in severe fibrotic lesions showed a significant increase relative to that in mild lesions. In contrast, no difference was detected in the numerical densities of CD68+ interstitial macrophages standardized by interstitial numerical density [N A(CD68)/N A(int)] (B). There was no difference in the numerical densities of CD163+ alveolar macrophages [N A(CD163)/A A(air)] and interstitial macrophages [N A(CD163)/N A(int)] between mild and severe lesions (C and D). The values of the numerical densities described in the figure represent actual values multiplied by 103. ∗∗∗∗ p<0.0001. [file 1436236.f2.pptx]

## Slide 1
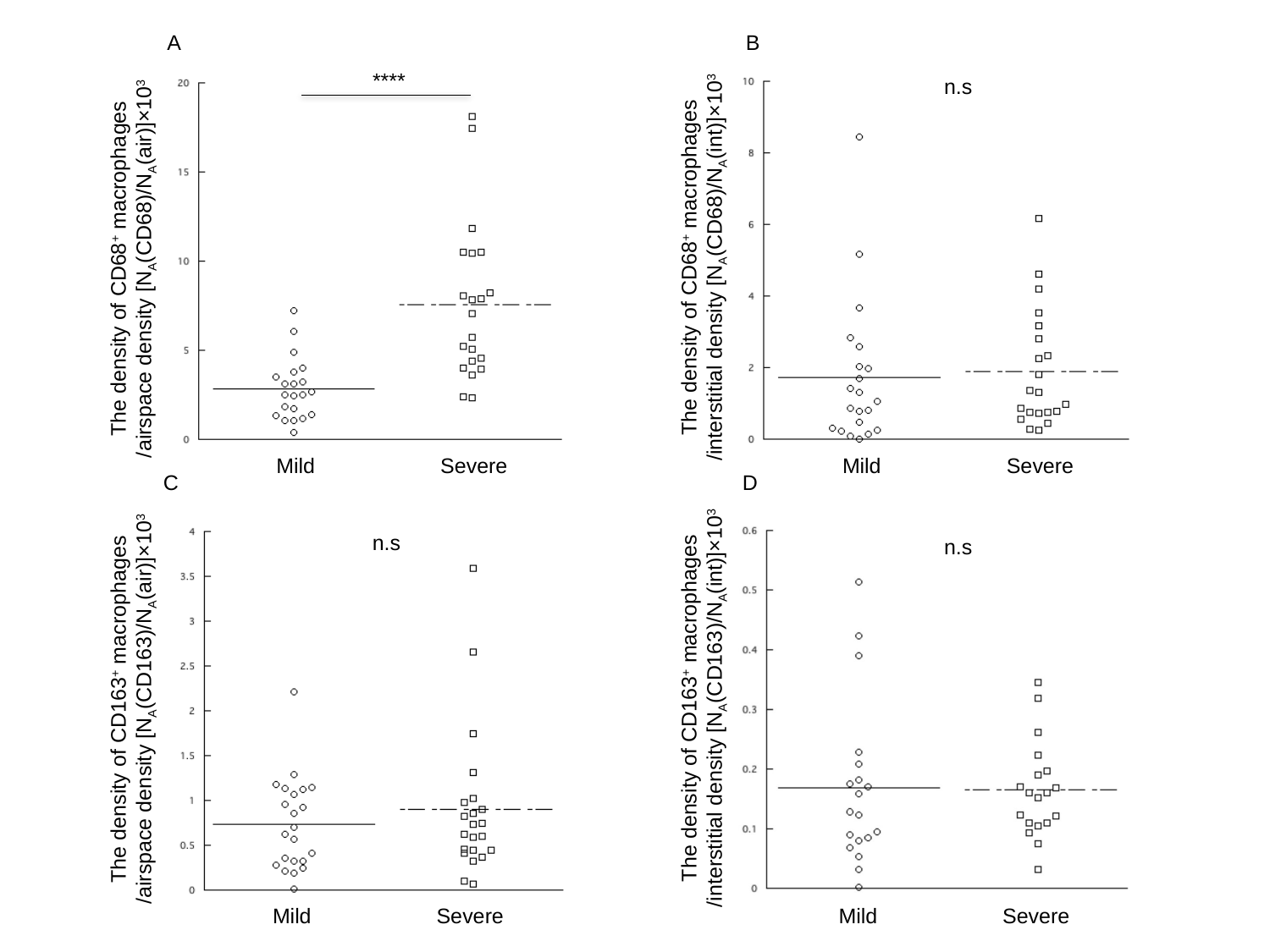

A
B
****
n.s
The density of CD68+ macrophages
/interstitial density [NA(CD68)/NA(int)]×103
The density of CD68+ macrophages
/airspace density [NA(CD68)/NA(air)]×103
Mild
Severe
Mild
Severe
C
D
n.s
n.s
The density of CD163+ macrophages
/interstitial density [NA(CD163)/NA(int)]×103
The density of CD163+ macrophages
/airspace density [NA(CD163)/NA(air)]×103
Mild
Severe
Mild
Severe
