# Supplementary material for: Distinct Profiles of CD163-Positive Macrophages in Idiopathic Interstitial Pneumonias
Source: J Immunol Res. 2018 Feb 4;2018:1436236. doi: 10.1155/2018/1436236 (PMC5817286; doi:10.1155/2018/1436236)
Supplement: Supplementary 3 — Figure E3: comparison of alveolar and interstitial densities of CD68+ and CD163+ macrophages among the 4 groups. The results of alveolar macrophages are similar to those of total macrophages in the case of CD68+ [N A(CD68)/N A(int)] (A), CD163+ macrophages [N A(CD163)/N A(int)] (B), and ratio of CD68+ macrophages to CD163+ macrophages [N A(CD68)/N A(CD163)] (C). The interstitial density of CD68+ macrophages [N A(CD68)/N A(int)] is similar to those of total macrophages, although CD68+ interstitial macrophages were not detected in 9 of 12 control cases (D). The results of CD163+ interstitial macrophages [N A(CD163)/N A(int)] are similar to those of alveolar and total macrophages (E). The interstitial ratio of CD68+ macrophages to CD163+ macrophages [N A(CD163)/N A(CD68)] showed a significant increase in IPF/UIP relative to that in the others (F). The values of numerical densities described in the figure represent actual values multiplied by 103. ∗ p<0.05, ∗∗ p<0.01, ∗∗∗ p<0.001, and ∗∗∗∗ p<0.0001. [file 1436236.f3.pptx]

## Slide 1
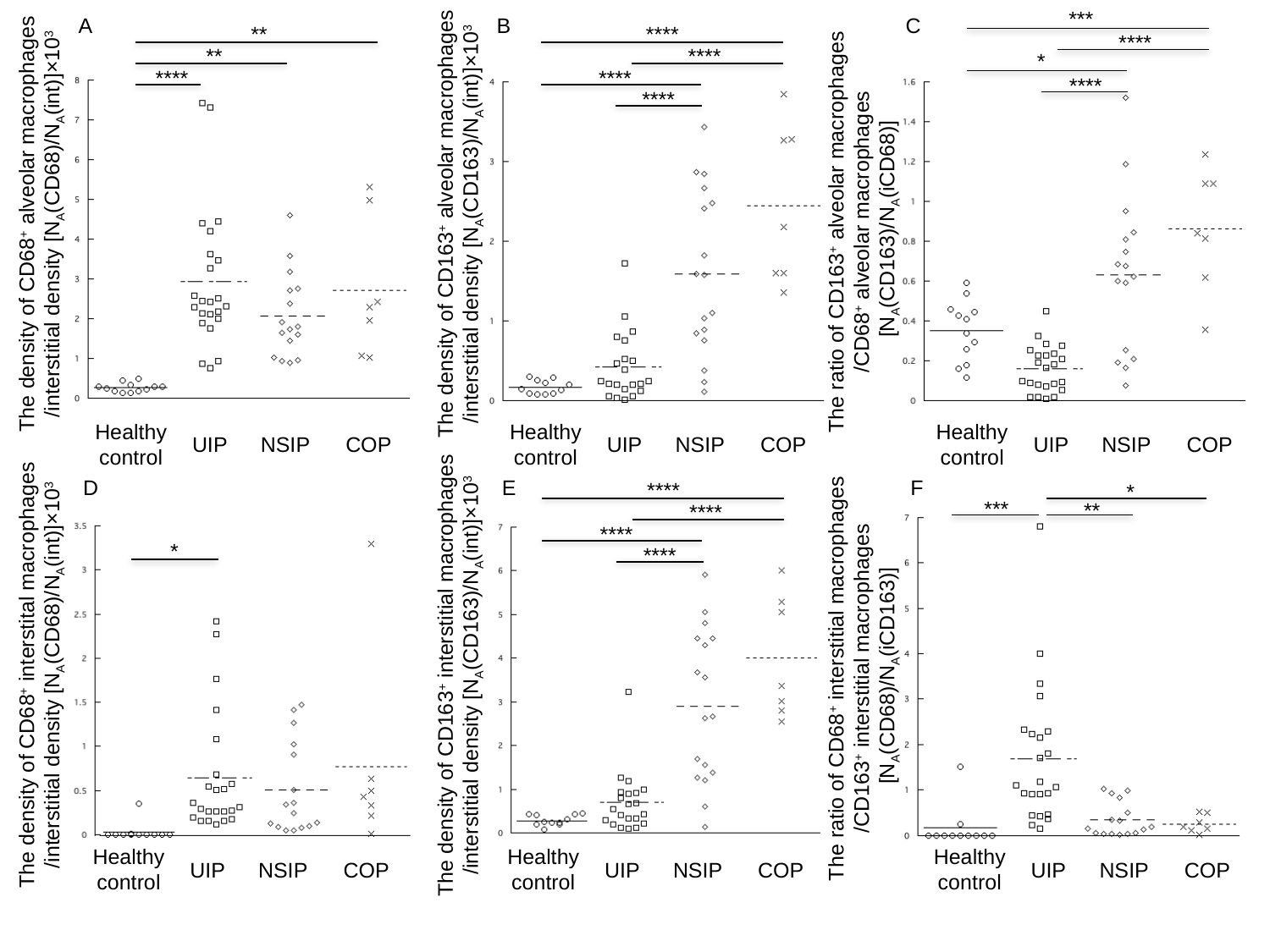

***
A
B
C
**
****
****
****
**
*
****
****
****
****
The ratio of CD163+ alveolar macrophages
/CD68+ alveolar macrophages
 [NA(CD163)/NA(iCD68)]
The density of CD68+ alveolar macrophages
/interstitial density [NA(CD68)/NA(int)]×103
The density of CD163+ alveolar macrophages
/interstitial density [NA(CD163)/NA(int)]×103
Healthy
control
Healthy
control
Healthy
control
UIP
NSIP
COP
UIP
NSIP
COP
UIP
NSIP
COP
D
E
F
****
*
***
**
****
****
*
****
The ratio of CD68+ interstitial macrophages
/CD163+ interstitial macrophages
 [NA(CD68)/NA(iCD163)]
The density of CD68+ interstital macrophages
/interstitial density [NA(CD68)/NA(int)]×103
The density of CD163+ interstitial macrophages
/interstitial density [NA(CD163)/NA(int)]×103
Healthy
control
Healthy
control
Healthy
control
UIP
NSIP
COP
UIP
NSIP
COP
UIP
NSIP
COP
