# Supplementary material for: Distinct Profiles of CD163-Positive Macrophages in Idiopathic Interstitial Pneumonias
Source: J Immunol Res. 2018 Feb 4;2018:1436236. doi: 10.1155/2018/1436236 (PMC5817286; doi:10.1155/2018/1436236)
Supplement: Supplementary 4 — Figure E4: comparison of numerical densities of CD68+ and CD163+ macrophages between nonsmokers and smokers. There was no difference in CD68+ and CD163+ macrophage densities and the ratio of CD163+ macrophages to CD68+ macrophages between nonsmokers and smokers in the normal control lungs (A–C). The ratio of CD163+ macrophages to CD68+ macrophages showed a decreasing trend in smoker patients with IPF/UIP relative to that in nonsmokers (F). A significant decrease was detected in smoker patients with NSIP relative to that in nonsmokers (H). The values of numerical densities described in the figure represent actual values multiplied by 103. ∗ p<0.05. [file 1436236.f4.pptx]

## Slide 1
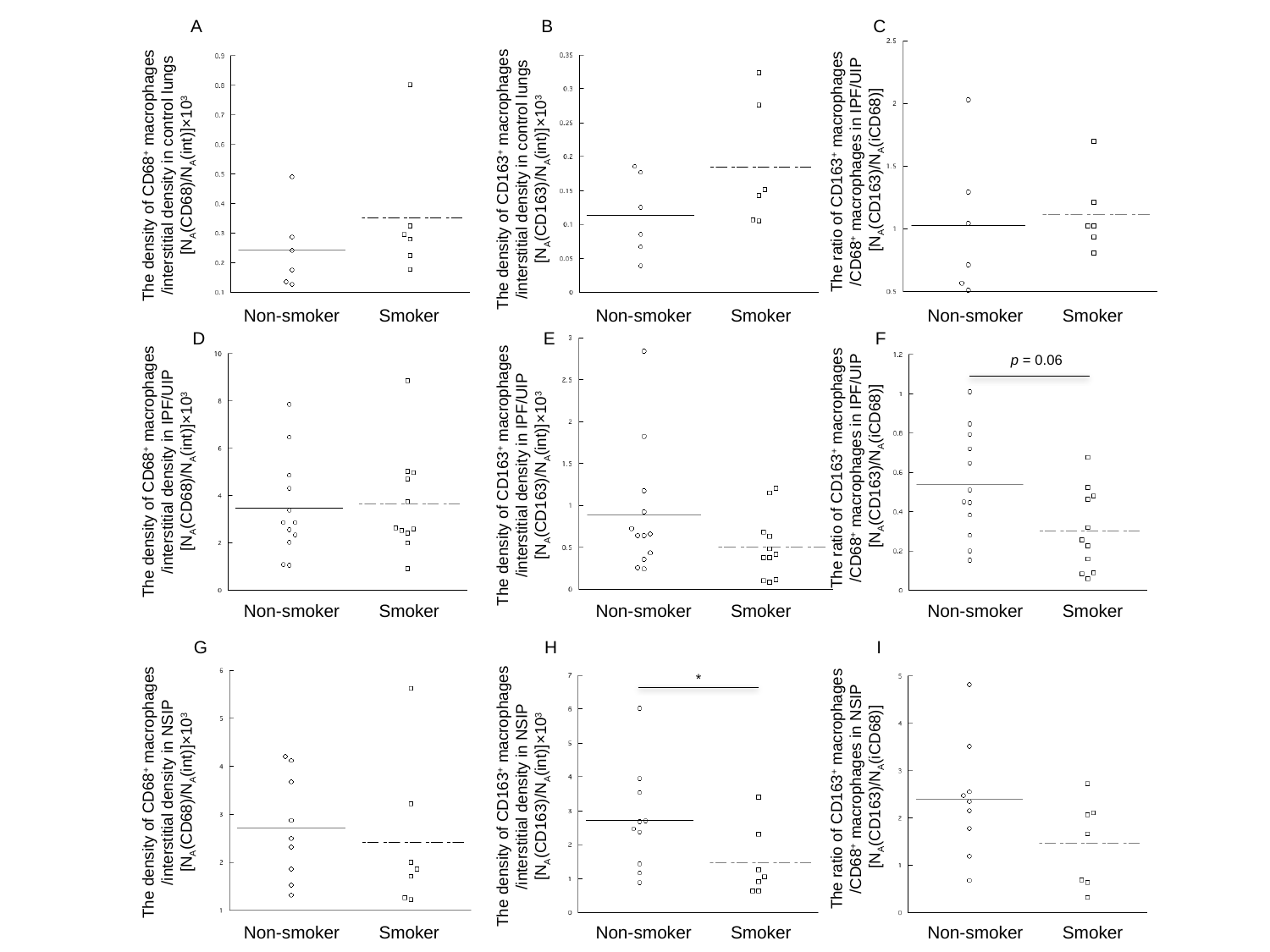

A
B
C
The ratio of CD163+ macrophages
/CD68+ macrophages in IPF/UIP
 [NA(CD163)/NA(iCD68)]
The density of CD68+ macrophages
/interstitial density in control lungs
[NA(CD68)/NA(int)]×103
The density of CD163+ macrophages
/interstitial density in control lungs
[NA(CD163)/NA(int)]×103
Non-smoker
Smoker
Non-smoker
Smoker
Non-smoker
Smoker
D
E
F
p = 0.06
The ratio of CD163+ macrophages
/CD68+ macrophages in IPF/UIP
 [NA(CD163)/NA(iCD68)]
The density of CD68+ macrophages
/interstitial density in IPF/UIP
[NA(CD68)/NA(int)]×103
The density of CD163+ macrophages
/interstitial density in IPF/UIP
[NA(CD163)/NA(int)]×103
Non-smoker
Smoker
Non-smoker
Smoker
Non-smoker
Smoker
G
H
I
*
The ratio of CD163+ macrophages
/CD68+ macrophages in NSIP
 [NA(CD163)/NA(iCD68)]
The density of CD68+ macrophages
/interstitial density in NSIP
[NA(CD68)/NA(int)]×103
The density of CD163+ macrophages
/interstitial density in NSIP
[NA(CD163)/NA(int)]×103
Non-smoker
Smoker
Non-smoker
Smoker
Non-smoker
Smoker
